# Dissemination of antimicrobial resistance in surface waters: surveillance of enterococci and coliform bacteria in Slovakia

**DOI:** 10.1007/s10123-026-00803-6

**Published:** 2026-03-25

**Authors:** Ivana Segéňová, Monika Hrušková, Lucia Bírošová, Júlia Koreneková, Petra Olejníková, Tomáš Mackuľak

**Affiliations:** 1 Faculty of Chemical and Food Technology, Department of Nutrition and Food Quality Assessment, Slovak University of Technology, Radlinského 9, Bratislava, 81237 Slovakia; 2 Faculty of Chemical and Food Technology, Institute of Biochemistry and Microbiology, Slovak University of Technology, Radlinského 9, Bratislava, 81237 Slovakia; 3 Faculty of Materials Science and Technology, Institute of Applied Informatics, Automation and Mechatronics, Slovak University of Technology, Ulica Jána Bottu 2781/25, Trnava, 91724 Slovakia

**Keywords:** antibiotic-resistant bacteria, antibiotic resistance genes, surface water, sediments, coliform bacteria, *E. coli*, enterococci

## Abstract

Antimicrobial-resistant faecal bacteria represent an increasing environmental concern in surface waters impacted by anthropogenic activities. Surveillance of enterococci and coliform bacteria, particularly *Escherichia coli*, is widely used to assess the dissemination of antimicrobial resistance in aquatic environments. These ecosystems may act as reservoirs of resistance determinants, where subinhibitory antibiotic concentrations promote their persistence and horizontal transfer, posing a potential public health risk.

This study aimed to survey antibiotic‑resistant coliform bacteria and enterococci in surface waters and associated sediments in the southern part of central Slovakia. Total coliforms were detected in all analysed water and sediment samples. *E. coli* was present in 50% of water samples and 75% of sediment samples, with sediments generally containing higher concentrations of both total coliforms and *E. coli*. Resistant coliform isolates predominantly exhibited multidrug resistance and strong biofilm‑forming capacity. Genes encoding broad‑spectrum β‑lactamases were detected in most isolates, with *bla*_*TEM*_ being the most prevalent. In isolates with reduced carbapenem susceptibility, *bla*_*NDM*_ was detected. Enterococci were detected in 63% of water samples and 75% of sediment samples, including *En. faecalis*, *En. faecium*, and *En. durans*. High resistance to ampicillin and vancomycin was observed, and three isolates carried the *vanA* resistance gene.

These findings highlight the role of surface waters in disseminating antimicrobial resistance in Slovakia and underscore the importance of continued surveillance in aquatic ecosystems.

**Supplementary Information:**

The online version contains supplementary material available at 10.1007/s10123-026-00803-6.

## Introduction

Antimicrobial resistance (AMR) is a biological phenomenon whose emergence and spread accelerated by anthropogenic factors, particularly the excessive and inappropriate use of antimicrobial agents (World Health Organization 2023a). A global analysis across 76 countries showed a 65% increase in total antibiotic (ATB) consumption between 2000 and 2015, and despite stewardship efforts, ATB use continued to grow, with a further 16% increase between 2016 and 2023 (Klein et al. 2018, 2024). This trend was intensified during the COVID-19 pandemic due to widespread prophylactic prescribing despite low rates of bacterial co-infection (Sério et al. 2023; Klein et al. 2024). A similar pattern was observed in Slovakia, where systemic antibacterial prescriptions, after a pre-pandemic decline, rose sharply following the onset of viral transmission and peaked in 2022 with nearly 40% year-on-year increase (National Health Information Center 2025).

The emergence and spread of AMR extend beyond clinical settings, with environmental transmission pathways recognised as critical drivers (Delair et al. 2024; Meradji et al. 2025). Aquatic ecosystems serve as major reservoirs and vectors of AMR, with wastewater identified as a key hotspot for antibiotic resistance genes (ARGs) and antibiotic-resistant bacteria (ARBs) (Birošová et al. 2014; Singh et al. 2022). Wastewater accumulates ATB residues from healthcare facilities, municipal sewage, pharmaceutical production and livestock farming, with all ATB classes detected at ng/L to mg/L concentrations (Birošová et al. 2014; Lenart-Boroń et al. 2020; Meradji et al. 2025). Sub-lethal ATB concentrations promote mutagenesis and horizontal gene transfer (HGT) within complex bacterial communities (Kusi et al. 2022). Other stress-inducing compounds present in wastewater, including pharmaceuticals, biocides, heavy metals, agrochemicals, nanomaterials and microplastics, further enhance selective pressure and AMR emergence (Meradji et al. 2025). Wastewater treatment is often insufficient, allowing many micropollutants to reach surface waters, confirmed by detection of over 200 pharmaceutical compounds in WWTP effluents worldwide (Lépesová et al. 2017; Štefunková et al. 2020; Sério et al. 2023). In addition, ARGs are also considered contaminants entering the environment through WWTP effluents as they can persist in treated wastewater at levels equal to or exceeding those in the inflow (Czekalski et al. 2015; Stange et al. 2016; Lai et al. 2021; Liu et al. 2025).

The occurrence of ARBs in aquatic environments—including surface waters, groundwaters, drinking waters and sediments—is increasingly common worldwide (Stange et al. 2016). Most bacteria in aquatic ecosystems exist in biofilms, which enhance survival and increase the HGT likelihood through high cell density and cohesion (Stiborová et al. 2018). HGT is not limited to closely related species and can occur between phylogenetically distant bacteria, turning non-pathogenic strains into reservoirs of ARGs (Baquero et al. 2008; Singh et al. 2022). This facilitates the transfer of ARGs to human pathogens, posing public health risks (Lenart-Boroń et al. 2020). Resistant pathogens may reach humans and animals via contaminated drinking water, recreational use of surface waters, or consumption of foods such as fish or irrigated vegetables (Meradji et al. 2025). To address these risks, the World Health Organization (WHO), in collaboration with FAO, UNEP and WOAH, proposed the “One Health” approach, aimed at reducing antimicrobial use and environmental contamination while limiting the development and spread of resistance (Singh et al. 2022; World Health Organization 2023b). The approach also emphasizes comprehensive research into AMR prevalence in aquatic ecosystems (Liu et al. 2025).

Given the increasing AMR problem in the environment, this study focused on the surveillance of resistant faecal indicators—coliform bacteria and enterococci—in Slovak surface waters and corresponding sediments. Antibiotic-resistant isolates were further characterized by susceptibility profiling, detection of ARGs and resistance mechanisms such as efflux pump overproduction, and evaluation of biofilm-forming capacity.

## Materials and methods

### Characterization of surface water sampling sites

**Fig. 1:**
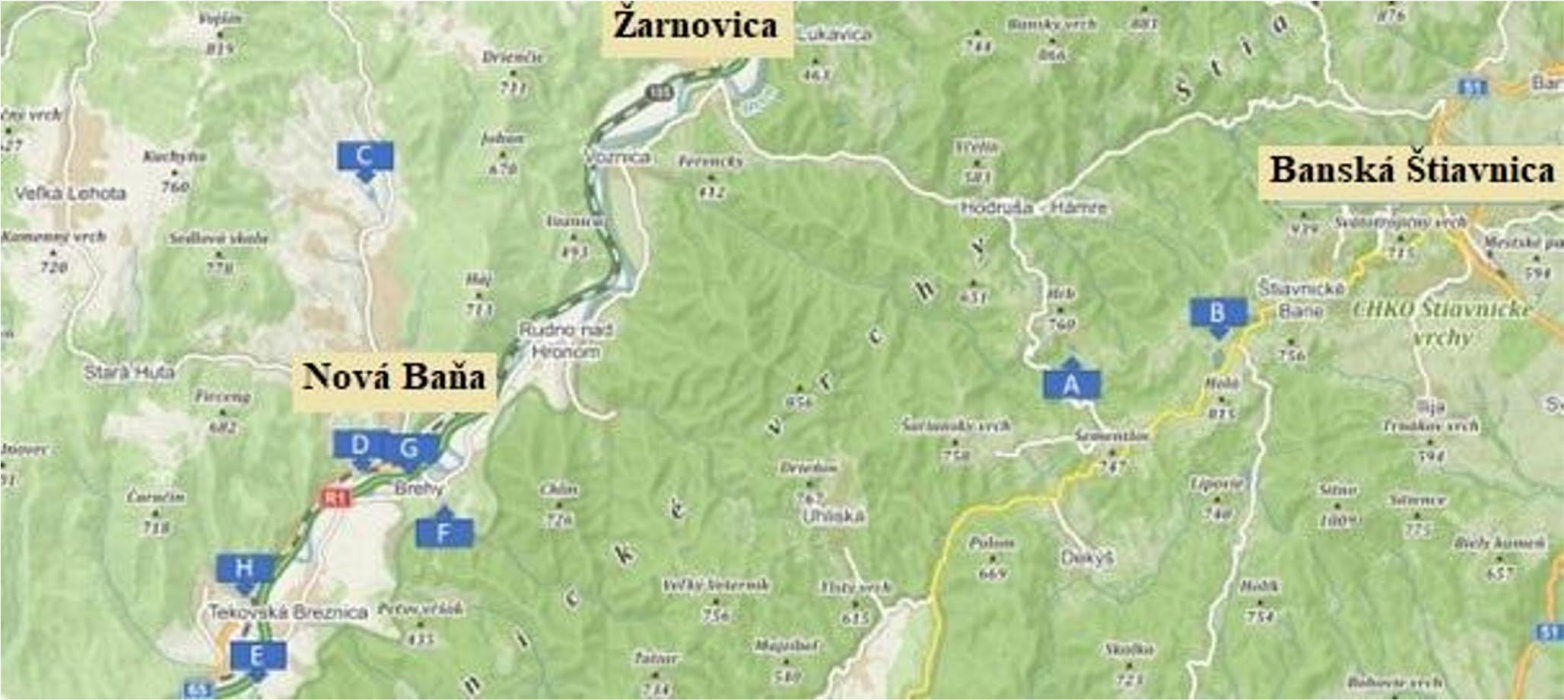
Locations of surface water sampling sites and corresponding sediments in the southern part of Slovakia. Stagnant surface waters: **A **– Kopanický tajch, **B **– Malá Richňava, **C **– Tajch Nová Baňa; **D **– Cibuľkové pleso, **E **– Luftov; Flowing waters: **F **– municipal stream Brehy; **G **– Hron river; **H **– Hron’s resting branch

The surface water samples originated from the southern part of central Slovakia and include flowing surface waters (*n* = 3) and stagnant surface waters (*n* = 5). The sampling was carried out with water and corresponding sediment as well. Water (*n* = 8) and sediment (*n* = 8) samples were collected in summer of 2018 (July-September) by point sampling along the shoreline of the water bodies into sterile bottles (500 mL each) and the samples were cooled and immediately transferred into the laboratory for microbiology analysis within 3 h. Spot sampling was performed in accordance with the standard STN EN ISO 19458:2007 (75 7770), *Water quality – Sampling for microbiological analysis*. A map of the sampling sites is shown in Fig. 1.

The sampling sites can be classified as:

- Stagnate surface waters: water reservoirs Kopanický tajch (A), Malá Richňava (B), Tajch Nová Baňa (C). These are artificially constructed water areas, which were previously used in the mining industry; lakes: Cibuľkové pleso (D), Luftov (E). They are located on the outskirts of the town of Nová Baňa near the Hron River, with possible groundwater seepage.
- Flowing surface waters: municipal stream overflowing a small village Brehy (F), Hron river (G), Hron’s resting branch (H). The municipal stream (F) flows directly through the village Brehy and is contaminated by domestic sewage from different households.

All sampling sites (A-H) are used as fishing grounds, with the majority of them classified as carp fisheries. Some water areas (A, B, C, G) are also widely used for recreational purposes in the summer seasons.

### Determination of total and resistant coliform bacteria and enterococci

In surface water and sediment samples, coliform bacteria (CFB) and *E. coli* were detected using Chromocult Coliform agar (VWR, Darmstadt, Germany), with incubation at 37 °C for 24 h. Simultaneously, enterococci (ENC) were determined using Slanetz and Bartley agar (BioLife Italiana srl., Milan, Italy), with incubation at 40 °C for 48 h. Water and sediment samples were serially diluted (10-fold) in physiological saline (8,5 g NaCl (Lach-Ner, Czech Republic)/1000 mL distilled water) and appropriate dilution was aseptically spread onto agar plates in 0.1 mL aliquot. In case of low bacterial count detection, the determination was repeated by filtering 10 mL of the sample through a GH polypro membrane (0,2 μm; Pall Corporation, USA).

For the detection and enumeration of resistant CFB, *E. coli* and ENC, agar media supplemented with antibiotics (ATBs) were used. In case of CFB and *E. coli*, ATBs as ampicillin (Amp), gentamicin (Gen), ciprofloxacin (Cip), chloramphenicol (Chl) and tetracycline (Tet) were used. In case of ENC, ATBs as Amp, Gen, Cip and vancomycin (Van) were used. Antibiotic concentrations (Table S1) were selected in accordance with MIC breakpoints defined by the European Committee for Antimicrobial Susceptibility Testing (EUCAST) (European Committee on Antimicrobial Susceptibility Testing 2018), except ATB Tet, where concentration defined by the Clinical and Laboratory Standards Institute (CLSI) (2018) was used as it is not established by EUCAST.

Each experiment was conducted in three parallels. The results were expressed as the mean ± standard deviation. Differences between water and sediment samples were assessed using Student’s *t*-test, with statistical significance set at *p* < 0.05. Data were treated with a one-way ANOVA test and a least significant difference of 95%.

### Isolation and identification of resistant bacteria

Resistant CFB, *E. coli* and ENC were inoculated on Mueller Hinton agar (BioLife Italiana srl., Milan, Italy) to obtain separate and pure colonies. Identification was performed via a Matrix–Assisted Laser Desorption/Ionization–Time of Flight (MALDI–TOF) mass spectrometer (Bruker, Germany) according to (Krahulcová et al. 2021). Single, fresh bacterial colonies were transferred onto a steel target plate (Bruker Daltonics Inc., Billerica, MA, USA) and overlaid with 1 µL of matrix. The matrix was prepared as saturated solution of α-cyano-4-hydroxycinnamic acid (Bruker Daltonics Inc., Billerica, MA, USA) in solution of 50% acetonitrile and 2.5% trifluoroacetic acid. The bacterial colony embedded in the matrix was allowed to dry at room temperature and the steel target plate was then inserted into the MALDI-TOF. For identification analysis, an AutoFlex I TOF-TOF apparatus (Bruker Daltonics Inc., Billerica, MA) and MALDI Biotyper software version 2.0 (Biotyper Library v.3.0; Bruker Daltonics Inc.) were used. Species-level identification was defined as a logarithmic score of > 1.9.

### Detection of ATB susceptibility

#### Macro-dilution method assay

Identified resistant bacterial isolates were detected for cross-resistance to various ATBs by a plate dilution drop method according to (Lépesová et al. 2018). Representants of different ATBs groups were used. In case of resistant CFB and *E. coli*, Amp (penicillins), Gen (aminoglycosides), ceftazidime (Cef; cephalosporins), Cip (fluoroquinolones), Chl (amphenicols), Tet (tetracyclines) (Sigma-Aldrich, Germany) and meropenem (Mer; carbapenems) (Cayman Chemical Company, USA) were used. In case of ENC, Amp, Gen, Cip and Van (glycopeptides) (PanReac Applichem, Spain) were used. *Enterococcus* spp. isolates with reduced susceptibility to vancomycin were additionally tested against teicoplanin using commercial ATB discs (Oxoid, UK). Each experiment was conducted in three parallels. The growth of coliform bacteria was evaluated visually after 24 h of incubation at 37 °C and, in the case of enterococci, after 48 h at 40 °C.

#### Microtiter plate assay

Bacterial isolates identified as Mer-resistant in *Macro-Dilution Method Assay* (2.4.1) were additionally evaluated with commercial microtiter plates (the Microlatest MIC assay; Erba Lachema, Czech Republic). Each microtiter plate contained a total of 24 ATBs. Overnight bacterial cultures were prepared in Mueller-Hinton broth (MHB; Biolife Italiana srl., Milan, Italy) and adjusted to a turbidity of 0.5 McFarland in sterile physiological saline. Subsequently, 100 µL of the bacterial suspension was inoculated into each well of the microtiter plate containing various antibiotics at different concentrations. Bacterial growth was evaluated spectrophotometrically (λ = 630 nm) after ~ 20 h of static incubation at 37 °C using a microplate reader (BioTek, USA). Testing and result interpretation were performed according to the manufacturer’s instructions.

### Detection of efflux pumps overproduction

Overproduction of efflux pumps was detected using Ethidium Bromide (EtBr) agar cartwheel method, with slight modifications (Martins et al. 2013). The detection of overproduction of efflux pumps was performed according to (Lépesová et al. 2020). Overnight bacterial cultures were prepared in MHB (Biolife Italiana srl., Milan, Italy) and adjusted to a turbidity of 0.5 McFarland in sterile physiological saline. Bacterial suspensions were inoculated onto MHA (Biolife Italiana srl., Milan, Italy) supplemented with EtBr (Serva, Germany). Experimental evaluation was performed visually under UV light, using the fluorescent properties of EtBr. As a negative control, *E. coli* (CCM 3988) was used for CFB, and *Staphylococcus aureus* (CCM 3953) for ENC, both obtained from the Czech Collection of Microorganisms in Brno. The experiments were repeated three times and ran in triplicates.

### Detection of biofilm formation

The ability to form biofilm and its quantitative assessment were previously described by (Lépesová et al. 2018). Biofilm formation was evaluated by measuring the absorbance at 570 nm using a microplate reader (BioTek, USA). Biofilm producers were classified into four categories–weak, medium, strong and very strong–according to (Taniguchi et al. 2009). As a positive control of biofilm production, *Pseudomonas aeruginosa* (CCM 3955) obtained from the Czech Collection of Microorganisms in Brno was used. Each experiment ran in six parallels and was repeated three times. Student’s *t*-test was applied for statistical analysis.

### Antibiotic resistance gene detection

Single and multiplex polymerase-chain-reaction (PCR) was used to determine ARGs. In case of CFB and *E. coli* isolates, genes encoding β-lactamases TEM, SHV, OXA and CTX-M group 1 and 2 (Dallenne et al. 2010), tetracycline resistance genes *tetA*, *tetE* (Ng et al. 2001) and gene encoding carbapenemases NDM, VIM, KPC and OXA-48 (Poirel et al. 2011) were examined. In case of ENC isolates, prevalent resistance gene *vanA* encoding resistance to vancomycin was examined (Depardieu et al. 2004). The sequences of the primers used are listed in Table 1.

**Table 1:** ARGs investigated by single and multiplex PCR assay in resistant isolates

ARGs	Primer		DNA Sequence 5´→ 3´	AS (bp)	AT (°C)	Source
*bla*_TEM_	MultiTSO-T_for	CATTTCCGTGTCGCCCTTATTC		800	60,5	(Dallenne et al. 2010)
	MultiTSO-T_rev	CGTTCATCCATAGTTGCCTGAC				
*bla*_SHV_	MutliTSO-S_for	AGCCGCTTGAGCAAATTAAAC		713		
	MutliTSO-S_rev	ATCCCGCAGATAAATCACCAC				
*bla*_OXA_	MutliTSO-O_for	GGCACCAGATTCAACTTTCAAG		564		
	MutliTSO-O_rev	GACCCCAAGTTTCCTGTAAGTG				
*bla*_CTX−M_	MultiCTXMGp1_for	TTAGGAARTGTGCCGCTGYA		688	60	
	MultiCTXMGp1-2_rev	CGATATCGTTGGTGGTRCCAT				
	MultiCTXMGp2_for	CGTTAACGGCACGATGAC		404		
	MultiCTXMGp1-2_rev	CGATATCGTTGGTGGTRCCAT				
*bla*_NDM_	Ndm_for	GGTTTGGCGATCTGGTTTTC		621	65	(Poirel et al. 2011)
	Ndm_rev	CGGAATGGCTCATCACGATC				
*bla*_VIM_	Vim_for	GATGGTGTTTGGTCGCATA		390		
	Vim_rev	CGAATGCGCAGCACCAG				
*bla*_KPC_	Kpc_for	CGTCTAGTTCTGCTGTCTTG		798		
	Kpc_rev	CTTGTCATCCTTGTTAGGCG				
*bla*_OXA−48_	Oxa-48_for	GCGTGGTTAAGGATGAACAC		438		
	Oxa-48_rev	CATCAAGTTCAACCCAACCG				
*tetA*	TetA_for	GCTACATCCTGCTTGCCTTC		210	53,6	(Ng et al. 2001)
	TetA_rev	CATAGATCGCCGTGAAGAGG				
*tetE*	TetE_for	AAACCACATCCTCCATACGC		278		
	TetE_rev	AAATAGGCCACAACCGTCAG				
*vanA*	EA1(+)	GGGAAAACGACAATTGC		732	65	(Depardieu et al. 2004)
	EA2(-)	GTACAATGCGGCCGTTA				

AS-amplicon size, AT-annealing temperature

The reaction mixture was prepared according to Table S2 and thoroughly vortexed. A DNA template was added to the reaction mixture as a pure colony from each resistant isolate. The total reaction volume was 50 µL for single PCR and 25 µL for multiplex PCR. The reaction mixture enriched with the pure colony of resistant isolate was inserted into the thermocycler (Mastercycler personal Eppendorf, Hamburg, Germany). The following conditions were applied: initial denaturation at 94 °C for 20 min.; cooling to 72 °C with a hold step while adding polymerase; denaturation at 94 °C for 40 s.; annealing at the appropriate temperature (AT) for 1 min; extension at 72 °C for 1 min and 30 s; and the final elongation step at 72 °C for 10 min.

After the initial denaturation (20 min.), the polymerase was added to the reaction mixture. For single PCR, Hot Start Taq DNA polymerase (5U/µl; Biotechrabbit, Germany) was used at a volume of 0.5 µL. In the case of multiplex PCR, Multiplex PCR Master Mix (Biotechrabbit, Germany) polymerase was used in a volume of 25 µl. Annealing temperature was set according to Table 1. The steps of denaturation to extension were repeated 35 times for tetracycline resistance genes and 30 times for all other ARGs. PCR products were separated and visualized using gel electrophoresis on a 1.5% agarose gel prepared in TAE buffer. The gel electrophoresis was performed at 100 V for 1 h 45 min. The gel was then stained with Gel Red (Biotium, Fremont, CA, USA) in TAE buffer for 30 min.

To confirm the presence of specific resistance genes, positive controls were verified by sequencing. After PCR amplification, selected amplicons were purified using the GenUp™ PCR/Gel Cleanup Kit (Biotechrabbit, Germany). Purification followed the manufacturer’s protocol based on adsorption of nucleic acids to a silica membrane and subsequent elution via rapid spin‑column processing. Purified PCR products were then submitted for Sanger sequencing to the Department of Molecular Biology, Faculty of Natural Sciences, Comenius University in Bratislava, which performed the sequencing as an external service provider.

## Results and discussion

### Occurrence of antibiotic-resistant bacteria in the environment

In this study, samples originate from stagnant surface waters (*n* = 5) and flowing surface waters (*n* = 3). A total of 16 environmental samples of surface waters and corresponding sediments were further analysed. The sampling locations are generally used for fishing or recreational activities. Coliforms, *E. coli* and ENC were examined as indicator organisms of faecal contamination in the samples (Pachepsky And Shelton 2011). Results of microbial analyses are summarized in Table 2.

**Table 2:** Counts of total and resistant CFB, *E. coli* and ENC in environmental samples (log CFU/mL for water, log CFU/g for sediment)

		A			B			C			D		
		water	sediment	ss	water	sediment	ss	water	sediment	ss	water	sediment	ss
**Coliforms**	total	3.45 ± 0.15	4.56 ± 0.20	***	3.60 ± 0.14	3.40 ± 0.21	*	3.28 ± 0.23	6.30 ± 0.40	**	2.66 ± 0.22	4.38 ± 0.31	***
	A	3.18 ± 0.11	4.52 ± 0.31	**	2.90 ± 0.10	3.36 ± 0.18	**	2.10 ± 0.16	6.19 ± 0.37	***	2.13 ± 0.18	4.23 ± 0.29	***
	G	3.17 ± 0.11	4.38 ± 0.24	**	< 0.30	3.08 ± 0.20	**	1.96 ± 0.08	6.20 ± 0.38	**	1.84 ± 0.09	4.08 ± 0.16	***
	C	< 0.30	< 0.30	-	< 0.30	< 0.30	-	< 0.30	< 0.30	-	< 0.30	< 0.30	-
	Ch	< 0.30	3.24 ± 0.10	***	< 0.30	< 0.30	-	< 0.30	2.70 ± 0.20	**	1.08 ± 0.04	1.70 ± 0.20	*
	T	< 0.30	1.30 ± 0.08	***	< 0.30	< 0.30	-	< 0.30	3.60 ± 0.25	**	< 0.30	< 0.30	-
***E. coli***	total	< 0.30	2.48 ± 0.12	***	< 0.30	1.48 ± 0.05	***	1.04 ± 0.10	4.60 ± 0.30	***	1.62 ± 0.10	2.00 ± 0.18	*
	A	< 0.30	2.20 ± 0.21	**	< 0.30	1.30 ± 0.06	***	0.95 ± 0.04	3.05 ± 0.22	**	1.18 ± 0.12	1.60 ± 0.11	***
	G	< 0.30	2.18 ± 0.18	**	< 0.30	1.30 ± 0.04	***	0.85 ± 0.05	2.99 ± 0.24	**	1.28 ± 0.15	1.30 ± 0.07	-
	C	< 0.30	< 0.30	-	< 0.30	< 0.30	-	< 0.30	< 0.30	-	< 0.30	< 0.30	-
	Ch	< 0.30	< 0.30	-	< 0.30	< 0.30	-	< 0.30	2.65 ± 0.19	**	< 0.30	< 0.30	-
	T	< 0.30	< 0.30	-	< 0.30	< 0.30	-	< 0.30	1.30 ± 0.08	**	< 0.30	< 0.30	-
**Enterococci**	total	< 0.30	1.90 ± 0.09	***	0.70 ± 0.04	1.70 ± 0.10	**	1.00 ± 0.05	3.53 ± 0.21	**	1.68 ± 0.10	2.00 ± 0.22	*
	A	< 0.30	< 0.30	-	< 0.30	< 0.30	-	< 0.30	1.30 ± 0.09	**	< 0.30	< 0.30	-
	G	< 0.30	< 0.30	-	< 0.30	< 0.30	-	< 0.30	1.60 ± 0.08	***	< 0.30	< 0.30	-
	C	< 0.30	< 0.30	-	< 0.30	< 0.30	-	< 0.30	2.70 ± 0.16	**	< 0.30	1.30 ± 0.14	**
	V	< 0.30	< 0.30	-	< 0.30	1.70 ± 0.12	**	0.30 ± 0.02	2.28 ± 0.20	**	0.30 ± 0.02	< 0.30	-
		**E**			**F**			**G**			**H**		
		water	sediment	ss	water	sediment	ss	water	sediment	ss	water	sediment	ss
**Coliforms**	total	3.18 ± 0.28	4.20 ± 0.32	***	3.83 ± 0.27	4.11 ± 0.25	**	2.49 ± 0.18	5.60 ± 0.33	***	2.30 ± 0.13	4.20 ± 0.29	**
	A	2.68 ± 0.15	4.10 ± 0.28	*	3.65 ± 0.22	4.10 ± 0.20	***	1.68 ± 0.16	5.45 ± 0.30	***	2.00 ± 0.20	3.92 ± 0.25	*
	G	2.58 ± 0.16	4.08 ± 0.28	**	3.70 ± 0.15	3.93 ± 0.20	-	1.71 ± 0.20	5.35 ± 0.30	***	2.30 ± 0.19	4.05 ± 0.30	**
	C	< 0.30	< 0.30	-	1.00 ± 0.04	2.00 ± 0.18	**	< 0.30	2.23 ± 0.14	**	< 0.30	< 0.30	-
	Ch	< 0.30	< 0.30	-	3.09 ± 0.28	3.08 ± 0.23	-	0.30 ± 0.03	3.24 ± 0.21	**	< 0.30	< 0.30	-
	T	< 0.30	< 0.30	-	1.60 ± 0.08	< 0.30	***	< 0.30	1.85 ± 0.11	**	< 0.30	< 0.30	-
***E. coli***	total	< 0.30	< 0.30	-	2.79 ± 0.21	2.38 ± 0.22	***	0.48 ± 0.04	2.28 ± 0.19	**	< 0.30	< 0.30	-
	A	< 0.30	< 0.30	-	2.73 ± 0.25	2.00 ± 0.19	**	< 0.30	2.08 ± 0.23	**	< 0.30	< 0.30	-
	G	< 0.30	< 0.30	-	0.90 ± 0.05	1.70 ± 0.08	***	0.48 ± 0.06	2.26 ± 0.22	**	< 0.30	< 0.30	-
	C	< 0.30	< 0.30	-	0.30 ± 0.02	< 0.30	-	< 0.30	1.48 ± 0.12	**	< 0.30	< 0.30	-
	Ch	< 0.30	< 0.30	-	1.08 ± 0.08	1.30 ± 0.07	-	< 0.30	1.85 ± 0.17	**	< 0.30	< 0.30	-
	T	< 0.30	< 0.30	-	1.45 ± 0.10	1.30 ± 0.08	**	< 0.30	1.78 ± 0.15	**	< 0.30	< 0.30	-
**Enterococci**	total	< 0.30	< 0.30	-	1.48 ± 0.09	1.30 ± 0.10	-	1.61 ± 0.15	1.48 ± 0.10	-	< 0.30	< 0.30	-
	A	< 0.30	< 0.30	-	< 0.30	1.30 ± 0.11	**	< 0.30	< 0.30	-	< 0.30	< 0.30	-
	G	< 0.30	< 0.30	-	< 0.30	< 0.30	-	< 0.30	< 0.30	-	< 0.30	< 0.30	-
	C	< 0.30	< 0.30	-	0.48 ± 0.04	< 0.30	*	< 0.30	< 0.30	-	< 0.30	< 0.30	-
	V	< 0.30	< 0.30	-	< 0.30	< 0.30	-	< 0.30	< 0.30	-	< 0.30	< 0.30	-

A – Kopanický Tajch, B – Malá Richňava, C – Tajch Nová Baňa; D – Cibuľkové pleso, E – Luftov; F – municipal stream Brehy; G –Hron river; H – Hron’s resting branch; A – ampicillin, C – ciprofloxacin, G – gentamicin Ch – chloramphenicol, T – tetracycline, V – vancomycin, ss – statistical significance determined using Student’s *t*-test comparing water and sediment samples (0.05 > *p* > 0.01 *, 0.01 > *p* > 0.001 ** and 0.001 > *p* *** - not significant)

Total CFB were detected in all tested samples of surface waters (2.30–3.83 log CFU/mL) and corresponding sediments (3.40–6.30 log CFU/g) (Table 2). Among water samples, the highest total CFB count was detected in sample F, a municipal stream flowing through small village Brehy (3.83 log CFU/mL). Moreover, water sample F also contained CFB resistant to all tested ATBs. Importantly, ciprofloxacin- and tetracycline-resistant CFB were presented only in this water sample. The high abundance and diversity of antibiotic-resistant CFB likely results from inadequate wastewater infrastructure, as the stream is impacted by household sewage. Additionally, summer sampling may have contributed to the results, as lower flow volume could increase the concentration of bacteria in the sample F. The lowest total CFB count was detected in water sample H, representing a Hron´s resting branch (Table 2).

Ampicillin is a widely used ATB in Slovakia and many other countries, which has contributed to the development of resistance not only to this ATB but also to other beta‑lactams. Resistance mechanisms to ampicillin are readily transmitted between bacteria through HGT and aquatic environments provide favourable conditions for the dissemination of resistant strains. Many coliform bacteria exhibit intrinsic resistance to ampicillin, which is consistent with the high prevalence of ampicillin‑resistant coliforms observed across all water and sediment samples in this study. Gentamicin-resistant CFB were detected in all samples as well, except one (*n* = 15), namely a water sample obtained from the Malá Richňava reservoir (B). Chloramphenicol-resistant CFB were detected only in three water samples (D, F and G) (Table 2).

A statistically significant difference was observed in the numbers of total CFB between sediment and surface water samples (*p* < 0.05). Total CFB counts were higher in sediment samples compared to the corresponding water samples in 7 out of 8 cases. These results correlate with those reported by (An et al. 2002) where lake sediments contained comparatively higher numbers of CFB than the overlying water. Bacteria occurring in sediments can form biofilms, which provide a form of protection against adverse environmental conditions. In addition, other sediment properties, such as high nutrient content, also promote the survival and growth of bacteria (Meradji et al. 2025). Antibiotic resistance to the specific ATB observed in water samples was observed in corresponding sediment samples as well in all tested samples, except the municipal stream (F). In case of sample F, tetracycline resistance was recorded only in the water sample. On the other hand, antibiotic resistance detected in sediment samples was not always observed in corresponding water samples, specifically chloramphenicol-resistant CFB in samples A and C, and tetracycline-resistant CFB in samples A, C and G. Statistically significant differences were also observed for ampicillin- and gentamicin-resistant CFB between water and sediment samples (*p* < 0.05).

One of the main criteria determining water quality is the presence of *E. coli* (Chmiel and Lenart-Boroń 2019). According to the WHO, *E. coli* is considered a reliable indicator of faecal contamination in water, as it is exclusively of intestinal origin (Sasakova et al. 2018). In our study, the occurrence of *E. coli* was observed in half of the samples, in numbers of 0.48–2.79 log CFU/mL (Table 2). According to the Slovak Ministry of Health Decree no. 308/2012 Coll. on the requirements for water quality, water quality control and the requirements for operation, equipment of operating areas, premises and facilities at natural and artificial swimming pools, none of our water samples used for recreational purposes exceeded the limit values ​​for the occurrence of *E. coli*. Total or antibiotic-resistant *E. coli* were not detected in two samples, neither in water nor sediment (E, H) (Table 2).

In a study by Štefunková et al. (2020), E. *coli* was detected in 85% of analysed surface waters, including rivers and lakes in Slovakia (Štefunková et al. 2020). In the surface water samples analysed in our study (*n* = 8), the most frequently detected antibiotic resistance among *E. coli* was to gentamicin (samples C, D, F and G), followed by ampicillin (Table 2). Unlike many other coliforms, *E. coli* is not intrinsically resistant to ampicillin. However, the high proportion of ampicillin-resistant strains within the total *E. coli* populations indicates an extensive dissemination of resistance in the environment, likely driven by the acquisition of resistance genes from other bacteria, including intrinsically resistant species. Among the water samples (*n* = 8), ciprofloxacin- and tetracycline-resistant *E. coli* were detected only in sample F, which contained *E. coli* strains resistant to all tested ATBs.

In sediment samples, *E. coli* was detected in 6 cases, in the range of 1.48–4.60 log CFU/g (Table 2). Similarly to the water samples, ampicillin- and gentamicin-resistant *E. coli* were the most detected ones in sediments. Among the sediments, *E. coli* resistant to all tested ATBs were found in one sample – the river Hron (G). Hron river (G) is the second longest river in Slovakia and is contaminated along its length by a lot of industrial production, livestock farming, several household sewages or WWTPs. Tetracycline- and chloramphenicol-resistant *E. coli* were detected in three sediment samples (C, F and G) (Table 2).

A statistically significant difference was observed in the numbers of gentamicin-resistant CFB between sediment and surface water samples (*p* < 0.05). Additionally, total and antibiotic-resistant *E. coli* were present in sediments of sites where their presence in the corresponding water samples was below the detection limit (A, B, C and G). According to various studies, increased concentrations of *E. coli* in water samples are often a result of resuspension of water sediments, which may act as a source of contamination (Jeng et al. 2005; Pachepsky And Shelton 2011).

Like CFB, ENC are considered as indicators of faecal contamination. However, ENC tend to persist longer in the environment (Sasakova et al. 2018). Total and antibiotic-resistant ENC were not detected in two samples, neither in waters nor sediments (E, H) (Table 2). Total ENC were detected in 63% of water samples in the range of 0.70–1.68 log CFU/mL. A study conducted in Poland reported ENC in all surface water samples, with counts repeatedly exceeded the limits (Łuczkiewicz et al. 2010). The detected occurrence also corresponds to another study conducted in Slovakia, where total ENC occurred in 70% of surface water samples (Štefunková et al. 2020). Among the water samples, only ciprofloxacin- and vancomycin-resistant enterococci (VRE) were detected in one (F) and two samples (C, D), respectively (Table 2).

The total ENC count ranged between 1.30 and 3.53 log CFU/g in sediments (*n* = 6) (Table 2). Sediment sample C, water reservoir Tajch Nová Baňa, contained ENC resistant to all tested ATBs, likely due to nearby livestock farming and an insufficient sewerage network. This surface water reservoir is also widely used for recreational purposes during the summer season. Apart from this, ampicillin-, ciprofloxacin- and vancomycin-resistant ENC were detected separately in three samples–F, D and B, respectively.

Sample A contained total ENC in water sample, but not in the sediment sample as the only site with this pattern. Comparison of water samples and their corresponding sediments revealed that antibiotic-resistant ENC were predominantly present in sediments in higher numbers, except for sample D, where VRE were detected only in the water sample. The increased abundance of ENC in sediments is neither unique nor surprising, as several studies indicate that bacteria in sediment survive longer than those in the overlying water (Jeng et al. 2005; Yoneda et al. 2024; Sosah et al. 2025).

Regardless of the origin of antibiotic-resistant bacteria (ARBs), their presence in different parts of aquatic ecosystems increases the possibilities of exchanging genetic material between microbial communities and contributes to the dissemination of AMR. Additionally, aquatic ARBs may increase the incidence of human infection through ingestion of contaminated water, for example during recreational activities (Štefunková et al. 2020).

### Identification of resistant isolates obtained from environmental samples

A total of 65 resistant CFB isolates and 12 resistant ENC isolates were successfully identified using MALDI-TOF mass spectrometry. The colonies were randomly selected from various growth media supplemented with different types and concentrations of ATBs used for the initial environmental surveillance.

**Fig. 2:**
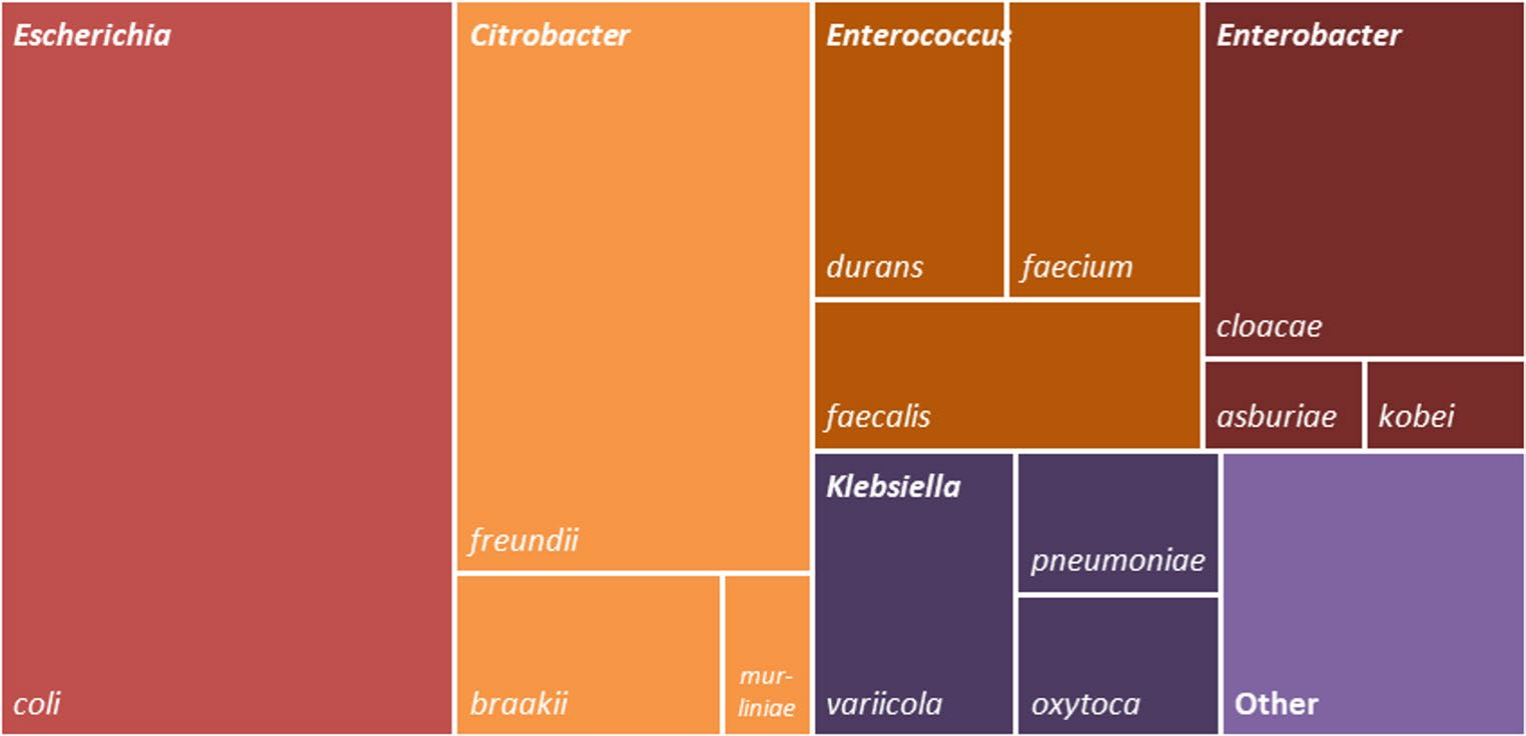
Identification of resistant isolates obtained from various environmental samples (surface waters and corresponding sediments). Other: *Raoultella ornithinolytica* (*n* = 2), *Raoultella planticola* (*n* = 1), *Lelliottia amnigena* (*n* = 1), *Leclercia adecarboxylata* (*n* = 1), *Kluyvera cryocrescens* (*n* = 1)

Figure 2 shows the distribution of the identified CFB isolates, with *E. coli* being the predominant species (*n* = 23), followed by representatives of the genus *Citrobacter* (*n* = 18), specifically the species *Citrobacter freundii* (*n* = 14), *Citrobacter brakii* (*n* = 3) and *Citrobacter murliniae* (*n* = 1).

Other identified genera included *Enterobacter* spp. (*n* = 10), *Klebsiella* spp. (*n* = 8), *Raoultella* spp. (*n* = 3), *Lelliottia amnigena*, *Leclercia adecarbolytica* and *Kluyvera cryocrescens* in this order (Fig. 2). Most of the resistant CFB originated from water samples (65%). In flowing waters (municipal streams) or stagnant surface waters, the presence of *E. coli* is most likely associated with anthropogenic contamination, such as discharges of municipal or agricultural waste and recreational activities. A high abundance of this species in water indicates faecal contamination, as the intestinal tract of humans and animals is considered the primary source of *E. coli* (Cho et al. 2020). Moreover, its presence may also indicate the occurrence of other pathogenic microorganisms (Truchado et al. 2018).

Twelve resistant ENC isolates were obtained from environmental samples, namely *En. durans*, *En. faecium* and *En. faecalis*, each represented by four isolates (Fig. 2). ENC were predominantly isolated from sediment samples (75%). The above-mentioned ENC species have repeatedly been detected in surface waters and sediments in previous studies (Cho et al. 2020; Dungan And Bjorneberg 2021).

### Evaluation of susceptibility profile and overproduction of efflux pumps in resistant isolates obtained from environmental samples

The antimicrobial susceptibility profiles of resistant CFB were characterized using seven ATBs from different classes: β-lactams (ampicillin), fluoroquinolones (ciprofloxacin), aminoglycosides (gentamicin), amphenicols (chloramphenicol), tetracyclines (tetracycline), cephalosporins (ceftazidime) and carbapenems (meropenem). Susceptibility was determined based on resistance breakpoints established by European (EUCAST) and American (CLSI) standards. American resistance breakpoints were generally higher and are denoted as R2 in Table 3, while resistance to the European breakpoints is marked as R1.

**Table 3:** Identification, susceptibility profile and overproduction of efflux pumps in resistant CFB and ENC isolates obtained from various environmental samples (surface waters and corresponding sediments)

**Sample origin**		**Isolate**	**Susceptibility testing**							**OV-EP**
			**A**	**C**	**G**	**Ch**	**T**	**Cef**	**Mer**	
**A**	S	*E. cloacae*	IR	R2	R2	S	R2	IR	S	+
		*E. asburiae*	IR	S	R1	S	R2	S	S	-
**C**	W	*E. coli*	R2	S	R1	S	R2	S	S	+
		*K. oxytoca*	IR	S	S	S	R2	S	S	-
	S	*E. coli*	R2	R2	R2	R2	R2	R1	S	-
		*K. variicola*	IR	R2	R2	R2	R2	R2	R1	-
		*K. variicola*	IR	R2	R2	S	R2	R2	S	+
		*K. variicola*	IR	R2	R2	S	R2	R2	S	-
		*K. variicola*	IR	R2	R2	R2	R2	R2	S	-
**D**	W	*E. coli*	R2	R1	R1	S	R2	S	S	-
		*E. coli*	R2	R2	R2	S	R2	S	S	-
		*E. coli*	R2	S	S	S	R2	R2	S	+
		*E. coli*	R2	S	R1	S	R2	S	S	-
		*E. coli*	R2	R2	R2	S	R2	R2	S	-
		*E. coli*	R2	S	S	R1	R2	S	S	-
		*E. cloacae*	IR	R2	R2	S	R2	IR	S	-
	S	*C. freundii*	IR	S	R1	S	R2	IR	S	+
**E**	W	*E. coli*	R2	S	S	S	S	R2	S	-
		*K. pneumoniae*	IR	R1	S	R1	R2	R1	S	+
		*K. pneumoniae*	IR	S	S	S	S	S	S	-
	S	*L. amnigena*	IR	S	R2	R2	R2	R2	S	-
		*E. cloacae*	IR	S	S	S	R2	S	S	+
		*R. planticola*	IR	R2	R2	R2	R2	S	S	+
		*R. ornithinolytica*	IR	R2	S	S	S	R2	S	+
		*K. cryocrescens*	IR	S	S	R2	S	S	S	+
**H**	W	*C. freundii*	IR	S	S	S	R2	IR	S	-
		*C. freundii*	IR	S	R2	R2	R2	IR	S	-
		*C. freundii*	IR	S	S	S	S	S	S	-
		*C. freundii*	IR	S	S	S	S	S	S	+
		*C. freundii*	IR	S	R2	R2	R2	IR	S	-
		*E. cloacae*	IR	R2	R2	R2	R2	IR	S	-
		*E. cloacae*	IR	S	R2	R2	R2	IR	S	-
**F**	W	*C. freundii*	IR	S	S	S	S	IR	S	-
		*C. freundii*	IR	R1	R2	R2	R2	IR	R2	+
		*C. freundii*	IR	R1	R2	R2	R2	IR	S	+
		*C. freundii*	IR	S	R2	R2	R2	S	S	-
		*C. freundii*	IR	S	R2	R2	R2	IR	S	+
		*C. freundii*	IR	S	S	S	R2	S	S	-
		*C. freundii*	IR	S	R2	R2	R2	IR	S	+
		*C. freundii*	IR	S	R2	R2	R2	IR	S	+
		*C. braakii*	IR	S	R2	R2	R2	S	S	+
		*C. braakii*	IR	S	R2	R2	R2	S	S	+
		*C. braakii*	IR	S	R2	R2	R2	IR	S	-
		*C. murliniae*	IR	S	R2	R2	R2	IR	S	+
		*E. coli*	R2	R2	R2	R1	R2	R1	S	+
		*E. coli*	R2	R1	R2	R2	R2	S	S	-
		*E. coli*	R2	S	S	S	R2	S	S	-
		*E. coli*	R2	S	R1	R1	S	S	S	-
		*E. coli*	R2	R1	R1	R1	S	S	S	-
		*E. coli*	R2	S	R1	S	R2	S	S	+
		*E. coli*	R2	S	R1	S	S	S	S	-
		*E. coli*	R2	R2	R2	R2	R2	R1	S	+
		*E. coli*	R2	S	R2	R1	R2	S	S	+
		*E. cloacae*	IR	S	R2	R2	R2	S	S	-
		*E. cloacae*	IR	R2	R2	S	S	IR	S	-
	S	*E. coli*	R2	R2	R2	S	R2	R2	S	-
		*E. coli*	R2	S	R1	R2	S	S	S	+
		*E. coli*	R2	S	R1	S	S	S	S	+
		*E. coli*	R2	S	R1	S	S	S	S	-
		*E. coli*	R2	R1	R2	R2	R2	S	S	-
		*E. kobei*	IR	R2	R2	S	R2	IR	R1	-
		*E. cloacae*	IR	S	S	S	R2	IR	S	+
		*K. oxytoca*	IR	R2	R2	R2	R2	S	S	+
		*R. ornithinolytica*	IR	S	S	S	S	R2	S	+
		*L. adecarboxylata*	IR	S	S	S	S	S	S	+
**Sample origin**		**Isolate**	**Susceptibility testing**							**OV-EP**
			**A**	**C**	**G**	**V**		**TC**		+
**C**	S	*En. durans*	R2	S	S	R2		S		+
		*En. durans*	R2	S	S	R2		S		-
		*En. durans*	R2	S	S	R2		S		+
		*En. durans*	R2	S	S	R2		S		-
		*En. faecium*	R2	S	S	R2		S		-
		*En. faecium*	R2	S	S	R2		S		-
		*En. faecium*	R2	S	S	R2		S		-
		*En. faecium*	R2	S	S	S		S		+
**F**	W	*En. feacalis*	R2	S	S	R2		S		+
		*En. feacalis*	R2	R1	S	S		S		+
		*En. feacalis*	R2	R1	R2	R1		S		+
	S	*En. feacalis*	R2	R1	R2	S		S		+

A – Kopanický Tajch, C – Tajch Nová Baňa; D – Cibuľkové pleso, E – Luftov; F - municipal stream Brehy; H – Hron’s resting branch; S – sediment, W – water; A – ampicillin, C – ciprofloxacin, G – gentamicin; Ch – chloramphenicol, T – tetracycline, Cef – ceftazidime; Mer – meropenem; V - vancomycin; TC - teicoplanin; S – sensitive; R1 – resistant to EUCAST breakpoint concentration, R2 – resistant to CLSI breakpoint concentration, IR – intrinsic resistance; OV-EP – overproduction of efflux pumps

The results in Table 3 indicate a high degree of AMR among the CFB isolates (*n* = 65) to the tested agents. The most prevalent resistance was observed for ampicillin. CFB possess intrinsic resistance to β-lactam ATBs through genes encoding the production of β-lactamases. An exception is ampicillin-susceptible *E. coli*, in which the resistance is acquired (Ruppé et al. 2015). Our findings, as presented in Table 3, demonstrate that *E. coli* isolates from all environmental samples exhibited resistance to ampicillin at concentrations specified by the CLSI standards (R2). High rates of ampicillin-resistant *E. coli* have also been reported in environmental samples in other studies, including those from Slovakia (wastewaters and surface waters) or in neighbouring countries such as the Czech Republic (surface waters) and Poland (rivers) (An et al. 2002; Štefunková et al. 2020; Lépesová et al. 2020).

Resistance to tetracycline was observed in 75% (*n* = 49) of the isolates (Table 3). For the detection of tetracycline resistance, only American criteria were applied, as European standards do not specify susceptibility testing for this class of ATBs within the family *Enterobacterales*, to which CFB belong. Tetracycline resistance was predominantly observed in *E. coli* (*n* = 16) and *Citrobacter* spp. (*n* = 15). Additionally, this type of resistance was detected in nine of ten *Enterobacter* isolates and in seven of eight *Klebsiella* isolates. Previous studies have frequently reported tetracycline-resistant *Citrobacter* spp., primarily originating from the faeces of various farm animals. This phenomenon is largely attributed to the extensive use of tetracyclines in animal husbandry, which exerts a sustained selective pressure favouring resistant strains (Chmiel and Lenart-Boroń 2019; Zhou et al. 2019). Conversely, the literature suggests that environmental isolates of *Citrobacter* spp. typically exhibit susceptibility to tetracycline (Figueira et al. 2012; Zhou et al. 2019).

Gentamicin resistance was detected in 72% (*n* = 47) of the resistant CFB isolates (Table 3). A substantial proportion of these isolates (75%) were able to tolerate the higher concentration specified by the American standard. In both Gram-negative and Gram-positive bacteria, the major mechanism of aminoglycoside resistance is enzymatic modification of the drug (Abdelaziz et al. 2021). The highest prevalence of gentamicin resistance was observed among *E. coli* (*n* = 19) and *Citrobacter* spp. (*n* = 13) isolates. A comparable incidence of gentamicin-resistant *E. coli* has been reported in wastewater isolates from Slovakia and the Czech Republic (Lépesová et al. 2020). However, several studies indicate a generally low rates of gentamicin resistance among members of the family *Enterobacterales* originating from surface waters or sediments (Figueira et al. 2012; Costa et al. 2019). For example, less than 5% of CFB isolated from the Danube River were reported as gentamicin-resistant (Kittinger et al. 2016). This finding is of clinical relevance, as the combination of β-lactams with gentamicin is commonly used in the treatment of hospitalized patients with bloodstream infections (Jakubovics And Grant Burgess 2015; Onorato et al. 2022). Resistance to chloramphenicol was detected in 49% (*n* = 32) of isolates, while resistance to ciprofloxacin was observed in 38% (*n* = 25) (Table 3).

In 2024, World Health Organization updated the list of drug-resistant bacteria posing the greatest threat to human health, in which third-generation cephalosporin-resistant and carbapenem-resistant *Enterobacterales* were classified as critical priority pathogens (World Health Organization 2024). In the present study, resistance to ceftazidime as a representative of third-generation cephalosporins was observed in 23% of isolates, while resistance to meropenem was detected in 3 isolates, specifically *K. variicola*, *C. freundii* and *E. kobei* (Table 3). These resistant isolates can therefore be considered putative producers of ESBLs or carbapenemases, respectively.

The antimicrobial susceptibility profile of the resistant *Enterococcus* isolates (*n* = 12) revealed a markedly high prevalence of resistance to several clinically important agents. Ampicillin resistance was observed in all isolates, followed by a substantial rate of vancomycin resistance, detected in 75% of isolates (Table 3). The elevated resistance to both vancomycin and ampicillin in *Enterococcus* species is particularly alarming, as these ATBs are crucial for the treatment of nosocomial infections caused by this genus and other Gram-positive pathogens (Echeverria-Esnal et al. 2023). Notably, vancomycin-resistant *En. faecium* is classified as a high-priority pathogen on the WHO Bacterial Priority Pathogens List, and three out of four *En. faecium* isolates in this study exhibited resistance at elevated CLSI breakpoints (R2) (Table 3) (World Health Organization 2024). Given that vancomycin represents a last-line treatment for severe Gram-positive infections, therapeutic failure may result in fatal outcomes. Consequently, the emergence and environmental dissemination of vancomycin resistance among opportunistic pathogens, with potential spillover into the wider community, constitutes a significant public health threat (Young et al. 2016).

In contrast, lower resistance rates were observed for ciprofloxacin (25%) and gentamicin (17%) (Table 3). The ciprofloxacin resistance rate identified in this study is comparable to the 22% reported by Łuczkiewicz et al. (2010) in streams and rivers in Poland, who also primarily identified *En. faecium* and *En. faecalis* strains, along with a smaller proportion of *En. durans*. However, their study reported a lower ampicillin resistance rate compared to our findings (Łuczkiewicz et al. 2010). In our environmental isolates, susceptibility testing also included teicoplanin, an ATB used in the treatment of severe Gram-positive infections, including those caused by methicillin-resistant *Staphylococcus aureus* (MRSA) (Vimberg 2021). All *Enterococcus* isolates were fully susceptible to teicoplanin (Table 3).

The term “multidrug resistance” (MDR) is internationally defined by the European Centre for Disease Prevention and Control (ECDC) and the Centers for Disease Control and Prevention (CDC) as acquired resistance to at least one antimicrobial agent in three or more distinct antimicrobial classes (Theuretzbacher 2013). In this study, 68% of CFB isolates from surface waters and associated sediments exhibited an MDR phenotype. *E. coli* accounted for the majority of these MDR strains (Table 4), with 78% (*n* = 18) of the resistant *E. coli* isolates (*n* = 23) classified as MDR. Among ENC, two isolates demonstrated MDR (Table 4), both originating from the municipal stream overflowing small village Brehy. The MDR ENC were identified as *En. faecalis*, with one isolate obtained from a water sample and the other from sediment (Table 3). The predominant MDR pattern involved resistance to three antimicrobial classes (46%), whereas in three cases resistance extended to six classes—specifically two *E. coli* strains and one *Klebsiella* spp. strain (Table 4).

**Table 4:** Number of MDR isolates obtained from environmental samples

Number of ATBs classes	Number of isolates						
	*E. coli* (*n* = 18)	*Citrobacter* (*n* = 12)	*Enterobacter* (*n* = 6)	*Klebsiella* (*n* = 6)	Lelliottia amnigena	Raoultella planticola	*En. faecalis* (*n* = 2)
**3**	7	10	4				1
**4**	5	1	2	4		1	1
**5**	4	1		1	1		
**6**	2			1			

Efflux pumps are integral transmembrane protein complexes inherently present in both Gram-positive and Gram-negative bacteria. The overproduction of efflux pumps represents a significant AMR mechanism, particularly noted for its prevalence among MDR bacteria (de Sousa Oliveira et al. 2016). In this study, efflux pump overproduction was identified in 45% of all resistant CFB isolates, mostly in *Citrobacter* spp. (*n* = 10) and *E. coli* (*n* = 8) (Table 3). This resistance mechanism was more frequently detected in isolates from sediment samples, where it was present in over half (56%) of all resistant CFB obtained from sediments. Conversely, efflux pump activity was not detected in the majority of CFB isolates (62%) from surface water.

Among ENC, efflux pump overproduction was detected in 8 out of 12 resistant isolates (Table 3). In Gram-positive bacteria, the contribution of efflux systems to resistance development is often considered indirect, as these mechanisms can modulate lipid efflux and thereby influence the efficacy of certain antimicrobial agents. For example, the *FarE* efflux system in *S. aureus* has been shown to regulate the secretion of lipids that protect the bacterial membrane from the membrane-targeting ATB rhodomyrtone. This mechanism, in which lipids act as a protective barrier, shares similarities with the extracellular release of membrane phospholipids that function as a decoy conferring resistance to daptomycin, a phenomenon observed in both resistant *S. aureus* and *En. faecalis* (Henderson et al. 2021).

### Evaluation of biofilm production in resistant isolates obtained from environmental samples

Biofilms are structured bacterial communities embedded in a self-produced extracellular polymeric matrix that facilitates attachment to surfaces and provides a protected microenvironment against hostile conditions (Jakubovics And Grant Burgess 2015). This enables bacteria within the biofilm to thrive in diverse ecosystems and promotes the horizontal transfer of mobile ARGs within the biofilm microbial community under certain conditions (Łuczkiewicz et al. 2010). Furthermore, in aquatic systems, biofilm formation and the presence of a sediment matrix can significantly enhance the transfer of resistance determinants between bacterial cells (Choudhury et al. 2012).

**Fig. 3:**
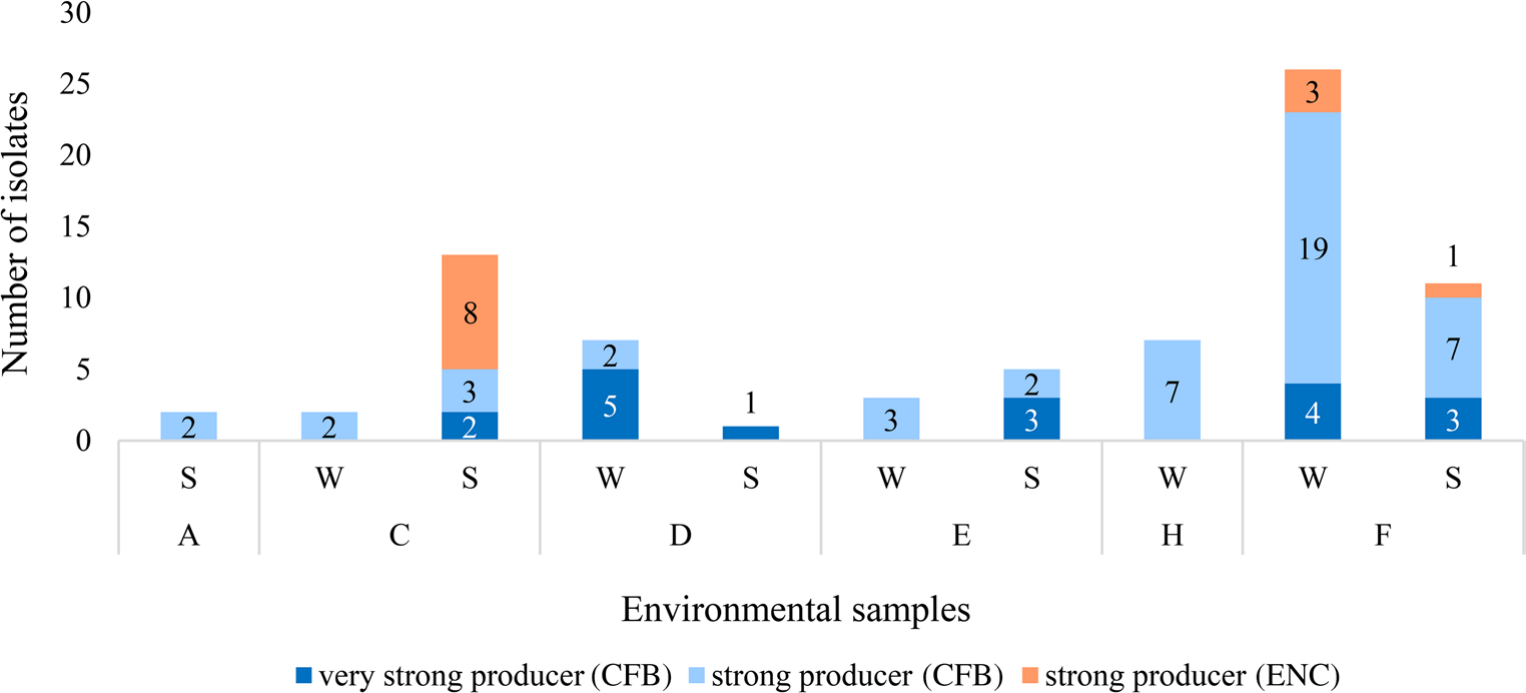
Biofilm formation rates of resistant isolates from various environmental samples (surface waters and corresponding sediments). A – Kopanický Tajch, C – Tajch Nová Baňa; D – Cibuľkové pleso, E – Luftov; H – Hron’s resting branch, F – municipal stream Brehy; CFB – coliform bacteria, ENC – enterococci; S – sediment, W – water.

Figure 3 illustrates the biofilm production capabilities of the resistant CFB and ENC isolates. The majority of resistant isolates from all environmental samples were classified as strong biofilm producers, including all ENC isolates and most of the CFB isolates (91%). Isolates exhibiting very strong biofilm production were CFB, namely *E. coli* (*n* = 7), *Citrobacter* spp. (*n* = 4), *Klebsiella* spp. (*n* = 3), *Enterobacter cloacae* (*n* = 2) and *Raoultella* spp. (*n* = 2).

### Detection of ARGs in resistant isolates obtained from environmental samples

Resistant CFB and ENC isolates were further evaluated for the presence of various ARGs, which were selected depending on the evaluated susceptibility profile of each isolate according to Table 3. In CFB, the following genes were detected: *bla*_*TEM*_, *bla*_*SHV*_ and bla_OXA_ encoding selected extended-spectrum β-lactamases (ESBLs), *bla*_*NDM*_ encoding a carbapenemase, and *tetA* gene encoding a tetracycline efflux pump. In ceftazidime-resistant CFB isolates, the *bla*_*CTX−M*_ gene was not detected. The trend of the abundance of ARGs was: *bla*_*TEM*_ > *bla*_*SHV*_ > *bla*_*OXA*_ = *tetA* = *bla*_*NDM*_. The most frequently detected gene *bla*_*TEM*_ (35%) was mainly detected in *Citrobacter* spp. (*n* = 12) and *E. coli* (*n* = 7) (Table 5).

**Table 5:** Occurrence of ARGs in resistant CFB and ENC isolates obtained from environmental samples

Sample origin		Resistance genes	β-lactamases			CPs	TRGs	VRG
		Resistant isolate	bla_TEM_	bla_SVH_	bla_OXA_	bla_NDM_	tetA	vanA
**F**	W	*E. coli* (*n* = 5)	5	1			1	
		*Citrobacter* spp. (*n* = 11)	10	1	1	1	1	
		*Enterobacter* spp. (*n* = 1)	1					
	S	*E. coli* (*n* = 2)	2					
		*Enterobacter* spp. (*n* = 2)		2		1		
		*K. oxytoca* (*n* = 1)			1			
		*L. adecarboxylata* (*n* = 1)						
**H**	W	*Citrobacter* spp. (*n* = 4)	2	3	1			
**E**	S	*Raoultella* spp. (*n* = 2)	2					
		*L. amnigena* (*n* = 1)	1				1	
	W	*K. pneumoniae* (*n* = 2)		2				
**C**	S	*K. variicola* (*n* = 3)		3		1		
		*En. durans* (*n* = 2)						2
		*En. faecium* (*n* = 1)						1
**D**	W	*E. coli* (*n* = 3)		3				

C – Tajch Nová Baňa; D – Cibuľkové pleso; E – Luftov; F – municipal stream Brehy; H – Hron’s resting branch; CPs – carbapenemases; TRGs – tetracycline resistance genes; VRG – vancomycin resistance gene; W – water; S – sediment

The *bla*_*TEM*_ resistance gene was predominantly detected in isolates obtained from the municipal stream overflowing the small village Brehy (F) (Table 5). This finding is likely a result of anthropogenic contamination, as wastewater discharge significantly influences not only the bacterial load (Table 2) but also the prevalence of ARGs. The *bla*_*TEM*_ gene is frequently observed in environmental water samples across Europe. For example, a study monitoring streams surrounding four major Swedish cities and Lake Mälaren, a key drinking water reservoir, also identified *bla*_*TEM*_ as the most prevalent ARG (Lai et al. 2021). Similarly, Amato et al. (2021) reported *bla*_TEM_ as the most widespread ARG in Spanish surface waters, detected in 96% of *E. coli* isolates (Amato et al. 2021).

The *bla*_*SHV*_ resistance gene was identified in 25% of isolates (Table 5). Most *bla*_*SHV*_ genes were detected in isolates from water samples, primarily *E. coli* (*n* = 4) and *Citrobacter* spp. (*n* = 4). Notably, 31% of all detected *bla*_*SHV*_ genes were found in *Klebsiella* spp. isolates from both water (E) and sediment (C) samples. It is important to note that *K. pneumoniae* possesses a chromosomally encoded *bla*_*SHV*_ gene that confers resistance to ampicillin via the production of penicillinase (Martin And Bachman 2018). In comparison, the *bla*_*SHV*_ gene has also been reported in German surface water samples, where it was detected in 16% of isolates (Stoll et al. 2012). Another study identified the *bla*_SHV_ gene in rivers and streams in Georgia, USA, using qPCR, with a detection rate of 9.4% (Cho et al. 2023).

CTX-M-type enzymes represent ESBLs that are rapidly disseminating among *Enterobacteriaceae* worldwide. They exhibit activity against extended-spectrum cephalosporins, including ceftazidime, although their activity is generally stronger against cefotaxime or ceftriaxone (Rossolini et al. 2008). More than half of our coliform isolates showed reduced susceptibility to ceftazidime; however, the majority were *Enterobacter* spp. and *Citrobacter* spp., which possess intrinsic resistance mediated by overexpression of chromosomal *ampC* β-lactamase genes (Paterson 2006; Sader et al. 2021). In contrast, in wild-type *E. coli* strains susceptible to cephalosporins, *ampC* expression is maintained at low levels due to degenerated promoter sequences. Cephalosporin resistance may be acquired through promoter mutations leading to AmpC hyperproduction or through acquisition of plasmid-mediated *ampC* genes originating from other enteric species (Pfeifer et al. 2010). However, the presence of these genes was not investigated in this study and warrants further examination. Nevertheless, the principal cause of third-generation cephalosporin resistance in *Enterobacteriaceae* remains ESBL production, particularly TEM- and SHV-derived enzymes that have evolved through mutations expanding their substrate specificity to third- and fourth-generation cephalosporins, including ceftazidime (Pfeifer et al. 2010). These enzymes were detected in the majority of ceftazidime-resistant isolates in our study. Moreover, overproduction of efflux pumps was observed in some of these isolates, which may further contribute to the ceftazidime resistance.

The *bla*_*OXA*_, *bla*_*NDM*_ and *tetA* ARGs were each identified in 5% of the resistant CFB isolates (Table 5). The *bla*_*OXA*_ gene was detected in *C. freundii* (*n* = 2) and *K. oxytoca* (*n* = 1) isolates obtained from the municipal stream overflowing the village Brehy (F) and from a Hron’s resting branch (H). In tetracycline-resistant CFB isolates, the *tetA* and *tetE* genes encoding tetracycline efflux pumps were investigated (Li et al. 2023). The *tetA* gene was detected in *E. coli* and *C. freundii* isolates from the municipal stream (F), as well as in a *L. amnigena* isolate from lake Luftov (E). While tetracycline ARGs have been reported in previous studies of lake and river samples, their prevalence in those studies was significantly higher (Khan et al. 2019; Shin et al. 2023).

The New Delhi metallo-β-lactamase (NDM) is a highly mobile, plasmid-encoded carbapenemase originally associated with hospital pathogens and hospital wastewater. However, the *bla*_*NDM*_ ARG has now been identified in numerous surface waters worldwide (e.g., Brazil, Canada, Vietnam, North Korea, China, and Japan), including the Danube River. These findings suggest that the *bla*_*NDM*_ variant is spreading rapidly, regardless of its primary source (Mills And Lee 2019; Zou et al. 2023). Environmental contamination, particularly with heavy metals, has been shown to facilitate the dissemination of ARGs (Gupta et al. 2022). In this study, the *bla*_*NDM*_ gene was detected in three resistant isolates: *C. freundii* obtained from water sample and *E. kobei* obtained from sediment, both originating from the municipal stream overflowing a village Brehy (F), and *K. variicola* from the Tajch water reservoir (C) (Table 5). Carbapenemase-producing *Enterobacterales* typically exhibit a MDR phenotype, leaving only a limited number of antimicrobial agents effective for treatment (Alvisi et al. 2025). This pattern was confirmed in all three aforementioned isolates, which were resistant to all tested antibiotics except *E. kobei*, which remained susceptible to chloramphenicol. Resistance to penicillins and cephalosporins is not surprising, as metallo-β-lactamases can hydrolyze nearly all β-lactams (Abdelaziz et al. 2021). In addition, ESBLs, which were also confirmed in these strains (*bla*_*SHV*_ in *E. kobei* and *K. variicola*, and *bla*_*TEM*_ in *C. freundii*), may further contribute to β-lactam resistance. In *C. freundii*, efflux pump overproduction has also been detected. Multidrug efflux pumps, which are widely distributed among resistant Gram-negative bacteria, can extrude a variety of ATBs, including β-lactams, fluoroquinolones, tetracycline and chloramphenicol (Abdelaziz et al. 2021). Given the sampling locations, the presence of *bla*_*NDM*_ gene is likely attributable to environmental contamination. At both sites, pollution has been documented due to an insufficient sewerage network and/or local livestock farming. Notably, the Tajch water reservoir (C) is an artificial water body originally constructed for mining purposes. During the summer season, it is widely used for recreational activities despite occasional official warnings. The *bla*_*NDM*_ gene has previously been identified in these bacterial species (Jung et al. 2020; Zhang et al. 2020).

Glycopeptide ATBs are used in the treatment of infections caused by Gram-positive bacteria. However, in bacteria of the genus *Enterococcus*, six types of glycopeptide resistance have been described, which can be distinguished based on the sequences of genes encoding ligase: *vanA*,* vanB*,* vanC*,* vanD*,* vanE* and *vanG* (Depardieu et al. 2004). The isolates were examined for the presence of the *vanA* gene, the predominant glycopeptide‑resistance determinant in ENC (Miller et al. 2014). Two isolates of *En. durans* and one isolate of *En. faecium* (Table 5) were positive for the *vanA* gene. In the remaining VRE isolates, reduced susceptibility to this ATB was likely associated with the presence of other resistance genes.

## Conclusion

Our surveillance of surface waters revealed that the majority of detected faecal bacteria exhibited antibiotic resistance. Despite the relatively small study area and limited number of sampling sites, we identified isolates resistant to all clinically relevant ATB groups tested, including carbapenems, which are classified within the *Watch* and *Reserve* categories of the WHO AWaRe classification of ATBs (World Health Organization 2023c). The presence of clinically significant resistant bacteria in surface waters underscores the critical role of aquatic ecosystems in the dissemination of antimicrobial resistance and highlights the urgent need for interventions aimed at mitigating contamination by both pharmaceutical residues and resistant microorganisms, in accordance with the principles of the “One Health” approach. A particularly concerning finding is that resistant isolates produce extended-spectrum β-lactamases and harbour multiple ARGs, which can be horizontally transferred to susceptible bacteria within aquatic ecosystem. Furthermore, the majority of isolates exhibited strong biofilm-forming capacity, enabling their persistence, proliferation, and the dissemination of resistance determinants.

This study demonstrates that surface waters and sediments in central Slovakia serve as reservoirs of antibiotic‑resistant coliform bacteria and enterococci. Several limitations should be acknowledged. Sampling was limited to a single season and a restricted number of sites, which may not fully reflect temporal and spatial variability. Taxonomic identification relied on selective media and MALDI‑TOF MS; although MALDI‑TOF provides rapid identification, its accuracy for environmental isolates may be constrained by database coverage. The absence of complementary 16 S rRNA gene sequencing constitutes an additional limitation. Furthermore, only selected resistance genes were analysed, without broader genomic approaches that could capture the full diversity and mobility of resistance determinants. Future research should expand monitoring across seasons and additional regions, and incorporate molecular sequencing and high‑resolution genomic methods to improve taxonomic resolution and deepen understanding of environmental resistomes and resistance dissemination pathways.

## Supplementary Information

Below is the link to the electronic supplementary material.

Supplementary Material 1 (DOCX 19.7 KB)

## Data Availability

Data of the current study are available from the corresponding author on reasonable request.
