# Supplementary material for: Dissemination of antimicrobial resistance in surface waters: surveillance of enterococci and coliform bacteria in Slovakia
Source: Int Microbiol. 2026 Mar 25;29(5):601–17. doi: 10.1007/s10123-026-00803-6 (PMC13260024; doi:10.1007/s10123-026-00803-6)
Supplement: Supplementary file 1 — Supplementary Material 1 (DOCX 19.7 KB) [file 10123_2026_803_MOESM1_ESM.docx]

**Supplementary materials**

**Table S1.** Concentrations of ATBs used for susceptibility/resistance determination

| **ATB** | **EUCAST** | **CLSI** | **ATB** | **EUCAST** | **CLSI** |
| --- | --- | --- | --- | --- | --- |
| coliforms | µg/mL | µg/mL | enterococci | µg/mL | µg/mL |
| ampicillin | (R>8) 9 | (R≥) 32 | ampicillin | (R>8) 9 | (R≥) 16 |
| gentamicin | (R>4) 5 | (R≥) 16 | gentamicin | (R>128) 130 | (R≥) 200 |
| ciprofloxacin | (R>0.5) 2 | (R≥) 4 | ciprofloxacin | (R>4) 5 | (R≥) 32 |
| chloramphenicol | (R>8) 9 | (R≥) 32 | vancomycin | (R>4) 5 | (R≥) 32 |
| tetracycline | - | (R≥) 16 |  |  |  |
| ceftazidime | (R>4) 5 | (R≥) 16 |  |  |  |
| meropenem | (R>8) 9 | (R≥) 5 |  |  |  |

ATB-antibiotic, EUCAST- European Committee for Antimicrobial Susceptibility Testing, CLSI- Clinical and Laboratory Standards Institute

Note: For the detection of resistant coliforms and enterococci based on MIC breakpoints defined by EUCAST, slightly increased concentrations of antibiotics were used, as the cut-off values for classification as "resistant" are given with a sign >.

**Table S2.** Preparation of reaction mixture for single and multiplex PCR assay

| Reagents | Single PCR Assay | | | | | Reagents | Multiplex PCR Assay | |
| --- | --- | --- | --- | --- | --- | --- | --- | --- |
|  | *bla*_CTX-M_ | | *bla*_NDM_ | *vanA* | |  | *bla*_TEM_, *bla*_SHV_, *bla*_OXA_ | *tetA, tetE* |
|  | volume (µL) | | | | |  | volume (µL) | |
| P1_fwd | 0,5 | | 1 | 0,25 | | P1_fwd | 0,5 | 0,5 |
| P1_rev | 0,5 | | 1 | 0,25 | | P1_rev | 0,5 | 0,5 |
| PCR buffer | 10 | | 10 | 10 | | P2_fwd | 0,5 | 0,5 |
| dNTPs | 1 | | 1 | 1 | | P2_rev | 0,5 | 0,5 |
| 50 mM MgCl_2_ | 2 | | 2 | 2 | | P3_fwd | 0,5 | - |
| WfMP | 36 | | 35 | 36,5 | | P3_rev | 0,5 | - |
|  |  |  | | |  | WfMP | 22 | 23 |

P1-P3_fwd-forward primers and P1-P3_rev-reverse primers (Metabion International AG, Germany), dNTPs- deoxyribonucleoside triphosphates, WfMP- deionized sterile water for molecular purposes (5 Prime, Germany).
